# Disclosure of Genetic Risk Factors for Alzheimer’s Disease to Cognitively Healthy Individuals—From Current Practice towards a Personalised Medicine Scenario

**DOI:** 10.3390/biomedicines10123177

**Published:** 2022-12-08

**Authors:** Samantha Galluzzi, Michela Pievani, Orazio Zanetti, Luisa Benussi, The Italian-DIAfN Working Group, Giovanni B. Frisoni, Emilio Di Maria

**Affiliations:** 1 Laboratory Alzheimer’s Neuroimaging & Epidemiology, IRCCS Istituto Centro San Giovanni di Dio Fatebenefratelli, 25125 Brescia, Italy; 2 Memory Clinic, IRCCS Istituto Centro San Giovanni di Dio Fatebenefratelli, 25125 Brescia, Italy; 3 Molecular Markers Laboratory, IRCCS Istituto Centro San Giovanni di Dio Fatebenefratelli, 25125 Brescia, Italy; 4 Laboratory of Neuroimaging of Aging (LANVIE), University of Geneva, 1205 Geneva, Switzerland; 5 Geneva Memory Center, Department of Rehabilitation and Geriatrics, Geneva University Hospitals, 1205 Geneva, Switzerland; 6 Department of Health Sciences, University of Genoa, 16132 Genoa, Italy; 7 University Unit of Medical Genetics, Galliera Hospital, 16128 Genoa, Italy

**Keywords:** Alzheimer’s disease, dementia, genetic risk, *APOE*, genetic testing, genetic counselling, health technology assessment, guidelines, review, Italy

## Abstract

Alzheimer’s disease (AD) is a genetically complex disorder. In addition to the relatively small number of pathogenic variants causing autosomal dominant AD, many others have been associated with the much more common sporadic form. The E4 allele of the Apolipoprotein E (*APOE*) is the first discovered genetic risk factor for AD. In addition, more than 70 genetic risk loci contributing to AD have been identified. Current guidelines do not recommend AD susceptibility genetic testing in cognitively healthy adults because the implications for clinical care are limited. However, secondary prevention clinical trials of disease-modifying therapies enrol individuals based on genetic criteria, and participants are often informed of *APOE* testing results. Moreover, the availability of direct-to-consumer genetic testing allows individuals to learn their own AD genetic risk profile without medical supervision. A number of research protocols for AD susceptibility genetic testing have been proposed. In Italy, disclosure processes and protocols beyond those developed for inherited dementia have not been established yet. We reviewed the literature on the current practice and clinical issues related to disclosing AD genetic risk to cognitively healthy individuals and provide suggestions that may help to develop specific guidelines at the national level.

## 1. Introduction

Alzheimer’s disease (AD) is a neurodegenerative disorder that is clinically characterized by progressive cognitive and functional impairment throughout the disease course. It is the most prevalent form of dementia (60–70%) and one of the major causes of disability and dependency among older people globally [1]. The personal, familial, and societal burden of the disease is overwhelming. Current treatments include drugs targeting cholinergic neurotransmission to enhance clinical symptoms in advanced stages, while only one disease-modifying drug has received conditional approval in the U.S. for earlier disease stages, but clinical benefits are uncertain [2]. Current research is increasingly focusing on the detection of the early phases of the disease, with a shift from cure to prevention and prediction.

The genetic underpinning of AD is complex and heterogeneous [3]. Beyond the relatively small number of rare pathogenic variants causing early-onset autosomal dominant AD, the search for genetic factors contributing to the more common late-onset AD has greatly evolved throughout the years, leading to the identification of up to 75 AD-associated genetic risk loci [4]. Rapid advances in AD genetics have huge implications for clinical research and personalised medicine. Genetic risk factors can be used to identify individuals at higher risk of developing AD for the purpose of clinical trial recruitment [5] and to define individualized risk profiling for preventive purposes in the community [6]. The ethical debate regarding the opportunity to disclose and communicate risk status (genetic and non-genetic) to cognitively healthy people participating in research studies is ongoing [7,8]. Moreover, the availability of direct-to-consumer (DTC) genetic testing allows individuals to learn their own AD genetic risk profile without medical supervision [9].

In this review, we address ethical and practical issues implied by assessing and disclosing AD genetic risk in cognitively healthy individuals. In the first part, we summarise the current state of AD genetics and the implementation of genetic risk prediction in clinical, research, and DTC settings. In the second part, we review the literature about and the current practices of AD genetic susceptibility testing disclosure across the above settings. In the third part, we discuss the ethical and practical implications of the disclosure of AD genetic risk test results, focusing on the Italian context. Based on our research experience in predictive genetic testing for inherited dementia [10,11], we comment on current limitations and summarise some hints that may form the basis to develop specific recommendations on this topic in Italy.

## 2. AD Genetics and Implementation of Genetic Findings

### 2.1. AD Genetics

Rare mutations in amyloid precursor protein (*APP*), presenilin 1 (*PSEN1*), and presenilin 2 (*PSEN2*) genes lead to early-onset familial AD, which represents ∼1% of AD cases. The discovery of these pathogenic variants provided important insights in the molecular mechanisms and pathways involved in AD pathogenesis and also led to valuable targets currently used in diagnosis and drug development [3].

The vast majority of late-onset sporadic cases of AD have no obvious familial aggregation, and it is considered to be multifactorial. However, genetic predisposition is strong, with estimated heritability being between 60 and 80% [12]. The genetic component in sporadic AD is heterogeneous. Starting from 1993, the e4 allele of Apolipoprotein E (*APOE*) was the only major gene known to increase AD risk, with a risk effect estimated to be threefold for e4 heterozygous carriers and 15-fold for e4 homozygous carriers [13,14]. The finding that the *APOE* e4 allele accounted for only 27% of the estimated disease heritability [15] led to a continued search for additional genetic risk factors. In the late 2000s, the advent of high-throughput genomic approaches significantly advanced the field, leading to the discovery of three new genetic risk factors [16]. Hence, over the last 12 years, up to 75 AD-associated genetic risk loci have been identified by genome-wide association studies (GWAS) and sequencing projects [4,17].

### 2.2. Implementation in Clinical Setting

The advancing knowledge of the genetic factors associated with AD might have the potential for clinical translation. However, the value of AD genetic risk factors in disease prediction in a clinical setting is limited, and current guidelines do not recommend genetic susceptibility testing in cognitively healthy adults [18]. The *APOE* e4 allele is neither necessary nor sufficient to cause the disease: up to 75% of the *APOE* e4 heterozygous carriers do not develop AD during life, and up to 50% of AD patients do not carry the *APOE* e4 allele [3]. Even in e4 homozygotes, penetrance is limited, suggesting that environmental factors [19] and other genetic variants [20] might moderate its effect. The clinical validity of genetic variants identified in GWAS is also limited, because each variant has a small individual effect [3]. The combination of genetic risk loci in polygenic risk scores can improve the prediction of an individual’s overall risk of developing the disease beyond *APOE* [5,21]. However, current models do not reach the accuracy required for clinical use [22] and were not validated by the mean of structured appraisals in the clinical context. Despite these limitations, the interest for the clinical applicability of genetic risk information is high. Recently, the European Task Force for Brain Health Services (BHS) envisioned new and innovative services aimed at implementing precision prevention programs targeting risk factors for primary and secondary dementia prevention [23]. Dementia risk profiling, including genetic risk, is part of the mission of BHS [6] and has already been adopted in Alzheimer’s Prevention Clinics in the U.S. [24]. In addition, clinical use of genetic risk information might increase in the near future, as *APOE* e4 has been shown to modify risks associated with amyloid targeting monoclonal antibodies [25].

### 2.3. Implementation in Research Setting

In clinical trials, the implementation of AD genetic risk information is already ongoing. In the context of AD prevention clinical trials, genetic risk information can be used to select research participants who are most likely to develop AD. For instance, *APOE* genotyping has been used to stratify risk at screening in the Anti-Amyloid Treatment in Asymptomatic Alzheimer’s (A4) study [26] and the Alzheimer’s Prevention Initiative (API) Generation Program [27], where the presence of at least one e4 allele was a core randomisation criterion. The goal was to identify individuals at higher risk for AD prior to more cost- and labour-intensive amyloid imaging, thus reducing screen failure rate and minimising costs, and ultimately to have a homogeneous population with the highest chance to benefit from treatment. Moreover, online research registries help to recruit individuals by matching characteristics of community volunteers with AD research trials for which they potentially qualify [28]. Participants from the registry can be invited to perform at-home *APOE* genotyping [29] or to share own *APOE* information with researchers [30] to facilitate referrals to targeted AD prevention studies.

### 2.4. Implementation in Direct-to-Consumer Setting

The progress in genomic research combined with the rapidly evolving field of personal genomics led private companies to offer DTC genetic testing, which may be defined as any DNA test for health or non-health (lifestyle) traits that are advertised and sold directly to the public via the Internet [31]. In 2017, the U.S. Food and Drug Administration approved genetic testing companies to offer DTC testing for late-onset AD risk [32]. Since then, the service has been made available also to some European countries [33], allowing interested individuals to know their AD risk profile, without medical supervision.

## 3. Protocols and Practice of AD Genetic Risk Disclosure

### 3.1. Clinical Setting

In the clinical setting of inherited AD, genetic counselling protocols are available to guide clinicians and geneticists in evaluating which individuals may benefit from genetic testing, according to the current guidelines [18]—such guidelines, in turn, could be developed thanks to the extensive research on genetic counselling and testing in Huntington’s disease, which provided the body of evidence for the updated recommendations [34]. In Italy, a research protocol for genetic counselling and testing in familial dementia was developed within the Italian Dominantly Inherited Alzheimer’s and Frontotemporal Network (IT-DIAfN) [35]. The IT-DIAfN protocol was elaborated through a consensus procedure among the participating expert centres in accordance with the current guidelines for genetic counselling and testing for late-onset neurodegenerative disorders [18,34]. The IT-DIAfN protocol consisted of: (i) at least 2 pre-test consultations, including a psychological assessment; (ii) blood sampling; (iii) disclosure of the genetic test result; and (iv) in-person follow-up. A multidisciplinary team of a geneticist, a psychologist or psychiatrist with expertise in counselling, and a neurologist or geriatrician with expertise in neurodegenerative diseases was involved in all sessions, except for the follow-up.

The IT-DIAfN protocol was implemented in a clinical research setting and showed to be feasible, acceptable, and safe in terms of the occurrence of catastrophic events [10]. Moreover, benefit on personal life and no detrimental impact on a broad range of psychological outcomes were demonstrated in individuals at-risk who underwent the counselling and testing procedure [11].

As AD genetic susceptibility testing is not recommended in clinical practice, a protocol of AD genetic risk disclosure is not available in clinics. The European Task Force for BHS developed practical guidelines for AD risk profiling that have to be implemented in the envisioned BHS [6]. They recommended the use of a dementia risk score as a measure of multiple modifiable risk factors and *APOE* e4 status testing as a measure of AD genetic risk, if resources allowed. If the resulting risk profiling indicated high dementia risk, the practical guidelines suggested testing for the common variant polygenic risk of late-onset AD as an additional optional investigation. The BHS Task Force stated that medical expertise (in neurology, geriatrics, or psychiatry) was necessary to interpret biological and genetic risk factors [23]. The collaboration of clinical geneticists was recommended in individuals with a family history of early-onset dementia, in whom rare variants conferring high AD risk can be investigated [23].

### 3.2. Research Setting

In the early 2000s, a series of clinical studies, the Risk Evaluation and Education for Alzheimer’s Disease (REVEAL), randomised individuals at risk for AD (i.e., asymptomatic adults having a first-degree relative with AD) to receive or not receive *APOE* results [36]. In the first REVEAL studies, information concerning risk was given to both groups by genetic counsellors using an extended protocol that followed current guidelines for AD predictive genetic testing [36] (Table 1).

In subsequent studies, the REVEAL investigators developed a condensed protocol, in which one of the two in-person pre-genetic test sessions was eliminated, using a mailed educational brochure instead of in-person education information; the blood-drawn visit was shortened and structured as a question-and-answer session, and administration of anxiety and depression scales was provided [37]. *APOE* disclosure was provided by a genetic counsellor or by a study physician who was a specialist in dementia but had received no formal training in genetic counselling. The condensed protocol did not impact the safety of disclosure relative to the extended protocol [37]. Lastly, a condensed research protocol that consisted of the telephone disclosure of genetic risk information was developed, but it failed to demonstrate non-inferiority for *APOE* e4 carriers [38] (Table 1).

When research participants in AD prevention clinical trials are selected based on genetic criteria, they are informed of their personal results. Therefore, research protocols for genetic counselling and disclosure have been developed, with a structure similar to that of condensed protocols in the REVEAL studies. In the API generation program [39], the process of *APOE* disclosure consisted of one-part in-person session, including education information and evaluation of psychological readiness for *APOE* disclosure; (ii) disclosure session with genetic counselling; and (iii) phone or in person follow-up 2–7 days after the disclosure, and until 12 months in *APOE* e4 homozygotes [39]. Providers of genetic counselling and disclosure should be trained clinical professionals with an advanced understanding of genetics and have experience with providing potentially sensitive medical results [39] (Table 1).

A similar condensed protocol was used in a study assessing safety and tolerability of *APOE* genotyping and disclosure in cognitively normal older adults from the Butler Alzheimer’s Prevention Registry [40]. The protocol included (i) an in-person visit to provide genetic education, to assess psychological readiness by clinical interview and standardized scales of depression, anxiety, and suicidal ideation, and to collect bio-samples for genotyping; (ii) an in-person *APOE* genotype disclosure visit; (iii) 3 remote follow-up visits until 6 months after disclosure. Study clinicians (board-certified neuropsychologists) conducted the study visits [40] (Table 1).

### 3.3. Direct-to-Consumer Setting

DTC genetic testing is marketed directly to the community, and the results are returned to users without the mediation of a healthcare provider. However, there is not a single DTC model, but a variety of formats and practices under which private companies or laboratories offer services, spanning from optional genetic counselling to tests delivered only through licensed professionals [41]. The American College of Medical Genetics and Genomics provided specific guidelines for DTC genotyping [42]. The European Society of Human Genetics published recommendations on clinical utility, laboratory quality standards, pre- and post-test counselling, and data privacy [43], but the legislation of DTC genetic testing is very fragmented in Europe, making unclear how such services are regulated [33]. In many EU Member States, the legislation requires the provisions of genetic testing to be conducted under medical supervision with genetic counselling, but applying the same laws in the commercial sector, outside of clinics or hospitals, is challenging [33].

## 4. Context-Sensitive Perspectives

### 4.1. How Can Genetic Risk Testing for AD Be Evaluated?

It is widely recognised that germline genetic testing, with respect to other diagnostic and prognostic procedures, is intrinsically featured by a unique peculiarity, which is the value of information for the offspring and relatives, as well as for the whole family in its largest definition. Due to this peculiarity, referred to as genetic exceptionalism, a number of protocols were developed in order to appraise the responsible transfer of genetic testing into clinical practice [44]. Most of them considered ethical, legal, and social issues (ELSI) as relevant domains to assess.

Some authors argued that the possible occurrence of the unfavourable psychological impact of predictive genetic testing was overestimated [45]; others underlined that non-health-related impacts (some personal, family, and societal effects) were investigated less often [46]. While the specific burden of predictive genetic testing for the individual, as measured by health outcomes, was extensively studied, the implications for the health care service were scarcely considered. In recent years, several efforts were made to close the gap in terms of formal assessment and proper technology transfer between genetic testing and other diagnostic and prognostic procedures. The newest recognised frameworks rely on the well-established health technology assessment (HTA) paradigm [44,47]. 

HTA is a multidisciplinary process that summarises information about the medical, social, economic, and ethical issues related to the use of health technology in a systematic, transparent, unbiased, and robust manner. Its aim is to inform the formulation of safe and effective health policies that are patient-focused and seek to achieve the best value [48]. Offering to patients and users health technologies that are able to convey health value [49] is the paramount basis of any universalistic public health system, such as most European health systems and notably the Italian one—the health value can be assumed as a key ethical principle shared by all community members.

### 4.2. Safety

A systematic review [50] of studies on clinical implications of predictive *APOE* genotyping for late-onset AD, published between 2008 and 2018, identified 5 studies addressing the safety of *APOE* disclosure and 80% (5/6) were from the REVEAL studies. They showed little psychological impact of *APOE* disclosure on depression, anxiety, and stress, with limitations due to exclusion of individuals with clinically significant anxiety and depression, high prevalence of female and well-educated individuals [51], and relatively short follow-up (1-year). It is noteworthy that test-related distress experienced by individuals receiving positive results from genetic susceptibility testing was low but similar to the experience of those receiving positive results for a deterministic mutation [52]. The other safety studies from the systematic review examined cognitive outcomes of *APOE* disclosure and showed adverse consequences on the perception of memory abilities and performance on objective memory tests [53]. In the U.S. context, *APOE* disclosure showed effects on advanced planning, which is why *APOE* e4 carriers were more likely to plan to purchase long-term care insurance than *APOE* e4 negative individuals [54].

Recent data on safety come from *APOE* genotype disclosure in the context of AD preventive clinical trials [55] and research registries [40]. A study in community-dwelling older adults from the Butler Alzheimer’s Prevention Registry showed no differences between e4 carriers and non-carriers on measures of depression or anxiety, but higher—even if subclinical—measures of stress in e4 carriers relative to non-carriers [40]. The study, as also in the REVEAL ones, included only individuals pre-screened for psychological readiness to learn AD genetic risk information. A qualitative study from the API Generation Program evaluated the impact of *APOE* disclosure on a broad range of outcomes [55]. The authors showed that knowledge of *APOE* results did not have a significant negative psychological impact, but it did have potentially adverse consequences on shaping self-perceptions and attitudes about memory, raised concern about stigma and discrimination in personal and professional relationships, and influenced financial planning, as well as having effects on family members [55].

The provision of DTC genotyping for genetic risk assessment without medical supervision and the potential psychological effects on consumers is a major concern and a subject of debate in research and professional community from its inception [56]. The limited literature showed that a relatively small percentage (3–4%) of consumers exhibited negative psychological effects related to such testing [57,58]. These figures increased up to 28% for higher penetrance genomic results, such as positive BRCA1/2 [59]. Data on DTC testing for AD risk are not available. Considering the stigma associated with AD [60] and the lack of an effective cure and robust preventive guidelines, it is reasonable to expect adverse reactions to AD genetic risk information in the absence of proper counselling, as suggested by case reports [61]. In a study aimed to identify predictors of adverse psychological experiences among DTC genetic test consumers who received personalized risk estimates for 23 common genetically complex diseases, the only medical condition that was predictive of clinically significant high anxiety before and after testing was having a family history of AD [62].

### 4.3. Users’ Perspective

General public interest in obtaining AD susceptibility testing is large, ranging from 50 to 75% [50]. Test-seeking behaviour was associated with having an immediate relative with AD [63,64], self-efficacy beliefs, and beliefs in the existence of preventive health behaviours [65]. Among DTC genetic testing users, 65% felt more in control of their health after receiving the results, and 59% said that the results would have an influence on their health management [66]. However, 60% of individuals would feel more comfortable if the option to consult a genetic counsellor was given with genetic testing [67]. Similar findings emerged in a recent qualitative study [68] that evaluated attitudes and motivations towards DTC genetic testing for late-onset AD in 31 young people aged 16 to 26 years—a generation towards DTC tests are heavily marketed [69]. The results showed that two-thirds of participants agreed for the test to be offered due to autonomy values (right to know and access). About one-third were interested in testing, primarily to gain self-knowledge regarding one’s health. However, face-to-face services were vastly preferred over the online option [68].

The use of enrichment strategies in AD prevention trials raised interest in evaluating the willingness of people from the community to learn their own *APOE* status and/or to share genetic information with researchers. A recent study [30] in 1312 enrolees of an online recruitment registry in the U.S. showed that only 7% used DTC genetic testing to learn their *APOE* genotype. From the others, 81% were interested in learning their genetic status. The willingness to share *APOE* information for study recruitment was >90% for both users and non-users [30].

### 4.4. The Italian Context

In Italy, data on users’ attitudes towards AD genetic susceptibility testing derives from the DTC setting. A sample of 152 users from an Italian private genetic company completed a survey addressing sociodemographics, motivations to test, health habits, psychological condition, perceived utility, and behavioural changes after results [70]. Only 1% of users underwent the genetic test to know their predisposition to AD—the most frequent analyses were nutrigenomics and oncoscreening. One-third of users keep directly in contact with the laboratory, while 60% preferred to manage procedures and results through their physicians [70]. Furthermore, compared with German DTC genetic testing users, Italian users were significantly more willing to share results with physicians and less with family members [71]. This finding suggests that Italian citizens assign physicians as the main source of information on health issues, including genetics, despite the increasing relevance of other information sources, such as mass media and the internet. What is unclear is the level of information the Italian doctors have about genetic matters, and how they are able to accompany their patients in the decision-making process [72,73,74]. An important drawback can be increasing referral to clinical genetics services and unnecessary costs for the National Health System.

## 5. Conclusions

Although several methodological issues are still to be solved, such as low predictive value and uncertainty of the risk estimates, it is reasonable to expect in the near future an increasing adoption of genetic risk testing of cognitively unimpaired individuals, especially when clinical trials of disease-modifying therapies eventually succeed, as hoped. Specific guidelines on counselling, disclosing genetic results and communicating risk are needed and require integration in the process of dementia care according to the national strategies [75].

In Italy, presymptomatic and susceptibility genetic testing is restricted for healthcare and healthcare-related research purposes and should be performed under medical supervision after obtaining written informed consent (Italian General Authorization for the Processing of Genetic data, 2014 [76]). Specific recommendations or guidelines on predictive genetic testing to determine the risk of developing AD have not been provided yet.

Here, we provide a few priority issues that may be further explored to elaborate a guide for the process of genetic risk assessment and disclosure. The key messages are summarised in Table 2.

### 5.1. Development of a Guidance

To ensure that guidance is adopted, the key principles for developing guidelines should be clarified; they should be based on the best available evidence of what works and what it costs; developed by independent and unbiased committees of experts, from across a range of health and social care professions, including lay members (people with personal experience of using health or care services, including careers or patients’ representatives); regularly maintained and updated in light of new evidence if necessary; and committed to advancing equality and diversity [77,78].

With regard to guidance for genetic risk disclosure in cognitively healthy individuals, the few hints reported here, as well as other relevant topics not reported for the sake of brevity, should be investigated and evaluated by an inclusive working group comprising all the relevant stakeholders—a provisional list should entail healthcare professionals and investigators with expertise in cognitive impairment, dementia, neuropsychology, genetic counselling, analytic and statistical procedures; policymakers and managers of health care services; representatives of families and communities, who may play a pivotal role in exploring the psychosocial domain and appraising the implications at the societal level.

A multidisciplinary working group may also suggest evidence-based suggestions on the most reliable and affordable analytic methods, for instance, as well on the effective format for report of results—i.e., categorical (increased or decreased risk with respect to the reference population) versus numerical estimates (risk percentage, with or without correction by age and sex).

It is easy to predict that *APOE* genetic testing will be overcome by other genetic risk estimation protocols, namely the use of polygenic risk scores, possibly combined with other risk variables [22]. Nevertheless, the development process for guidance on the established technologies may be a valuable exercise, which may eventually inform the regular update of the best evidence-based practice in the light of novel, well-established technologies.

### 5.2. Genetic Risk Communication

Risk communication is an integral part of genetic counselling and testing for inherited neurodegenerative diseases, including dementia, and the literature on this topic provides guidance for best practices. Within the context of clinical trial enrolment, risk disclosure is embedded in the research protocol, as already outlined. In the clinical context, while keeping in mind that AD susceptibility genetic testing is currently not recommended, evidence-based risk communication strategies aimed at personalised risk reduction are under development [6,79].

Genetic risk communication for sporadic AD is challenging because genetic variants are not causative, and personal risk perception can be misinterpreted. Therefore, special attention to emotional, familial, and sociocultural influences on the risk communication process is required [80], in line with principles and good practices of genetic counselling in neurodegenerative disorders [18,34]. From the patient-centredness perspective, any genetic counselling protocol should be focused on the individual view of his/her health status and risk, rather than on the actual risk estimates. In this perspective, genetic risk should not be communicated as a prominent and independent risk factor, but in the context of a personalised dementia risk profile, which takes into account other common predictors, such as age, social determinants of cognitive health, lifestyle, etc. [6]. Additional research is needed to establish the validity of dementia risk prediction models and their clinical utility in different populations.

### 5.3. Setting and Professionals

The studies examined clearly demonstrated the users’ attitude in favour of face-to-face services rather than the online option. Genetic testing is perceived as an option to acquire additional knowledge of one’s health, and therefore most users rely on the presence of healthcare professionals with specific expertise [65,66,67,68,69]. When the study protocols are focused on AD risk and prevention, although the majority of clinical research protocols are based in either geriatrics or neurology departments, study participants are basically enrolled from the community-dwelling population. According to the current guidelines and practice, the team must include, together with the clinical specialist(s) in charge, medical geneticists and/or genetic counsellors, neuropsychologists and/or clinical psychologists. As a result, the multidisciplinary team is in charge of the whole process of AD genetic risk assessment and disclosure—as opposed to one single professional. 

It can be argued that the implementation of the full genetic counselling and testing protocol developed for predictive testing in families with inherited AD [18,35] is demanding and expensive. Nonetheless, a minimum set of standard requirements should be guaranteed, also in clinical research protocols (by the way, within a clinical trial, the cost due to the implementation of a counselling protocol is a marginal share of the total cost).

### 5.4. Continuing Education

The need for education for all parties was apparent in the current literature. From the patient/consumer perspective, the great expectations linked to the development of molecular genetic testing may need to be moderated. A careful explanation should be provided (and its efficacy should be tested) clearly separating genetic testing with high predictive value (rare fully penetrant genetic variants) and genetic risk variants which alone have a limited predictive value [22]. Moreover, it should be reminded that there is no definitive evidence that individuals at high genetic risk are more likely to benefit from targeted dementia prevention intervention [6]. Educational strategies for the public may improve genomic literacy and increase abilities to make appropriate health decisions [31,76].

Multiple studies, especially in the Italian context, demonstrated the insufficient knowledge of healthcare professionals about the use of genomic technologies [72,73]. As far as capacity-building initiatives are advisable, the objective of educational tools for medical doctors, whether specialists or general practitioners, cannot be the achievement of the same skills envisaged for medical geneticists. Only multidisciplinary and multi-professional teams host the competence and expertise to deal with the multifaceted issues that may emerge in the course of a genetic risk assessment and disclosure pathway.

### 5.5. Patient-Centred Health Value

Ethical principles in healthcare—namely autonomy and non-directiveness—had been invoked as fundamental values in predictive genetic testing since the inception of presymptomatic genetic testing in neurodegenerative disorders (see [18,51] and articles quoted therein). In the context of AD genetic risk based on variants with low predictive value, there is no reason to consider autonomy less valuable—any eligible testee should be in the position to autonomously decide at any time whether to know or not to know her/his genetic risk for AD (in addition to the considerations made for Mendelian neurodegenerative diseases, the uncertainty related to the limited predictive value of *APOE* testing should be cautiously considered). An autonomous choice implies full information and time to decide, with the support of a specifically trained team. In clinical research, if a protocol does not allow the choice, eligible individuals should be aware that participation implies the genetic risk disclosure—or blindness, according to the study protocol.

Equity was not considered as an ethical dimension as well, though molecular analyses can be considered at risk, in terms of possible unfavourable impacts on inequalities in access to health technologies. Equity should be a priority for decision-makers in a universalistic national health system, which is the paradigm for the implementation of health care services in most European countries—undoubtedly in Italy. Therefore, equitable access to the highest attainable standard should be a goal for all healthcare providers. Moreover, not all that is affordable should be offered unless the attainment of the quality standard has been appraised for the fundamental domains of HTA.

The impact on equity may be an additional added value of a shared protocol for disclosing AD genetic risk. Even in the research setting, the highest standard of care should be equitably offered to all participants, regardless of the study location or source of funding.

### 5.6. Limitations and Final Considerations

We are aware that this review, including our considerations, stemmed from the inspection of the literature and suffers from many limitations. Notably, the literature search and the results extraction were not conducted using systematic procedures. Since our effort had been inspired by the application of AD risk disclosure in current clinical and research pathways, the newest methodologies, such as complex risk scores or artificial intelligence tools, were not considered. Finally, the present manuscript is far from covering all relevant issues connected with the disclosure of the genetic risk of AD in healthy individuals—e.g., genetic data protection was beyond the scope of the present work.

However, we did not find a large number of high-quality studies addressing the different issues here explored. Further research is needed—population studies and clinical trials—with rigorous design and controlled risk of bias. Research protocols should be equally accessible to the whole target population, including minority and less affluent individuals, and should be conducted in diverse cultural and national contexts, accounting for the local healthcare systems.

In conclusion, keeping in mind that the use of genetic risk estimations for AD is increasing, though at a slower pace than expected, it is worth promoting specific actions aimed at improving the current practice: (i) evidence should be accumulated, considering research questions that were rarely investigated; (ii) equity should be a continuous goal, leading to the design of more inclusive clinical studies; (iii) engagement in the clinical and research communities is pivotal to close the gap between expectations and practice.

## Figures and Tables

**Table 1:** Clinical guidelines and research protocols for AD genetic risk disclosure.

Protocol	Population	Genetic Variant	Delivery	Personnel	Pre-Test Evaluations	Structure
ACMG and NSGC guidelines [18]	Individuals with familial AD	*PSEN1, PSEN2, APP*	In-person, videoconference	Genetic counsellor	Neurologist, psychologist/psychiatrist	Two-part pre-test; one or more post-disclosure follow-up
The REVEAL study [36]	Individuals with first-degree AD relative	*APOE*	In-person	Genetic counsellor	Scales on depression, anxiety, stress	Two-part pre-test; three post-disclosure follow-ups
The REVEAL study [37]	Individuals with first-degree AD relative	*APOE*	In-person	Genetic counsellor or study physician	Scales on depression, anxiety	One-part pre-test; three post-disclosure follow-ups
The REVEAL study [38]	Individuals with first-degree AD relative	*APOE*	Telephone (genetic disclosure)	Genetic counsellor	Scales on depression, anxiety, stress	One-part pre-test; three post-disclosure follow-ups
API Generation Program [39]	Older individuals enrolled in the trial	*APOE*	In-person, telephone (follow-up)	Provider qualified per local regulations	Scales on depression, anxiety, suicidal ideation	One-part pre-test; one or more post-disclosure follow-up
Butler Alzheimer’s Prevention Registry [40]	Older individuals volunteering in the register	*APOE*	In-person, online (follow-up)	Board-certified neuropsychologist	Scales on depression, anxiety, suicidal ideation	One-part pre-test; three post-disclosure follow-ups

ACMG and NSGC: American College of Medical Genetics and National Society of Genetic Counsellors; REVEAL: Risk Evaluation and Education for Alzheimer’s Disease; API: Alzheimer’s Prevention Initiative; AD: Alzheimer’s disease.

**Table 2:** Disclosure of genetic risk factors for AD to cognitively healthy individuals—key messages and future perspectives.

Topic	Key Word	Key Message
Genetic risk communication	Counselling	Risk communication should be included in an integrated genetic counselling and testing procedure.
	Practice	Currently, testing genetic risk factors is not recommended in clinical practice.
	Research	In clinical research, risk disclosure should be embedded in the relevant research protocol.
	Patient-centredness	User-centred procedures are warranted.
	Personalised risk	The genetic risk should be interpreted and disclosed to the participant as part of a comprehensive individual risk for dementia.
Guidance	Guidelines	Evidence-based guidelines are warranted and should be developed by independent experts from across a range of health and social care professions, including lay members.
	Inclusiveness	All relevant stakeholders should be allowed to appraise the implications—or the risks and benefits—of disclosing genetic risk factors to healthy individuals.
	Innovation	Novel technologies should be timely evaluated by using structured assessment procedures (HTA).
Protocol	Setting	Users would prefer face-to-face services rather than remote consultations, as genetic testing is perceived as an option to gain knowledge on one’s own health; users rely on the presence of expert healthcare professionals.
	Multidisciplinarity	A multidisciplinary team should be in charge of the whole process of genetic risk assessment and disclosure.
.	Quality	The procedure should comply with acknowledged quality standards; proper resources should be allocated, also in clinical research protocols.
Education	Health education	Educational strategies for the public may improve genomic literacy and increase abilities to make appropriate health decisions.
	Medical education	Continuing education programmes for healthcare professionals about the clinical utility of genomic technologies are warranted.
	Interdisciplinarity	Multidisciplinary and multi-professional teams may guarantee the ability to deal with the multifaceted issues implied by genetic risk disclosure.
Health value	Autonomy	Enrolled individuals should be able to autonomously decide whether to know or not to know her/his genetic risk; the uncertainty related to the limited predictive value of currently available genotyping should be considered.
	Technology assessment	Structured assessments should be deployed to evaluate all domains of the genetic risk assessment and disclosure procedure—safety, effectiveness, economic and organizational issues, ethical, legal and social issues.
Perspectives	Evidence	Further research is needed—investigations featured by rigorous design and controlled risk of bias will contribute to accumulate knowledge; novel research questions may be considered.
	Equity	Research protocols should be equally accessible, including minority and less affluent individuals, and should be conducted in diverse cultural and national contexts.
	Engagement	The proactive attitude of the clinical and research communities will help closing the gap between expectations and practice; users and other relevant stakeholders may contribute to the development of appropriate pathways.

## Data Availability

Not applicable.
